# 30. SARS-CoV2 Reinfections in a University Teaching Hospital

**DOI:** 10.1093/ofid/ofab466.030

**Published:** 2021-12-04

**Authors:** Una Sutton, Judi Lynch, Niamh Fitzgerald, Guerrino Macori, Séamus Fanning, Jérôme Fennell

**Affiliations:** 1 Tallaght University Hospital, Dublin, Dublin, Ireland; 2 UCD School of Public Health, Physiotherapy & Sports Science, Dublin, Dublin, Ireland; 3 Trinity College Dublin, Dublin, Dublin, Ireland

## Abstract

**Background:**

The consequences of SARS-CoV2 reinfections for patients, healthcare workers and society are unclear. We reviewed the clinical, laboratory, and epidemiological characteristics of patients re-infected with genetically distinct strains of SARS-CoV2 identified by Whole Virus Genome Sequencing (WvGS).

**Methods:**

Cases were selected based on a positive SARS-CoV-2 Reverse Transcriptase Polymerase Chain Reaction (RT-PCR) test, clinical resolution, a negative interim test and a subsequent positive nasopharyngeal swab. Positive samples were prepared for sequencing by cDNA synthesis, tiled-PCR following the ARTIC protocol and amplicon sequencing using Illumina MiSeq platform. Raw reads were mapped to the reference sequence using bowtie and Samtools was used for variants calling and to generate the consensus sequences. Comparative sequence analysis was conducted by phylogenetic inference maximum likelihood method with RAxML using the multiple sequence aligned by MAFFT. Clades and variants were assigned respectively using Nextstrain and Pangolin COVID-19 lineage assigner (Figure 1). The clinical, radiological and laboratory data were collected from patient medical notes and laboratory information system.

**Results:**

Two cases of SARS-CoV-2 reinfection were detected by RT-PCR (patient 1 and 2). C_T_ values and strain variants are presented in Table 1. The time between detection of the first and second infection was 67 and 270 days respectively. WvGS confirmed that the second episodes were due to a genetically distinct strain of SARS CoV2. These reflected the dominant contemporaneous variants in circulation.

Both patients were immunocompromised from co-morbidities and medications. First and subsequent infections were minimally symptomatic. Both cases were associated with known hospital outbreaks. They passed away within 2 weeks of the second infection of unrelated causes.

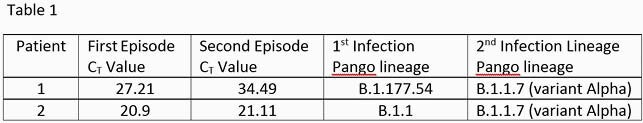

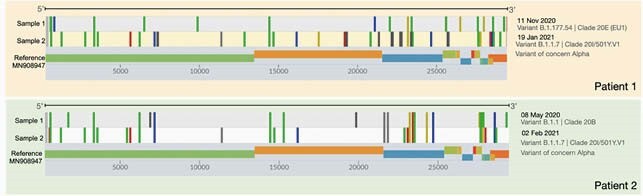

**Conclusion:**

Two patients in this study were diagnosed with a SARS-CoV-2 reinfection confirmed by WvGS. A common factor in these cases was immunocompromise. Where a previously infected patient test shows a new positive or an unexpected reduction in C_T_ value is observed, we recommend individual risk assessment to determine the timing of discontinuation of isolation and infection control precautions.

**Disclosures:**

**Jérôme Fennell, MB BCh BAO MSc PhD FRCPath FRCPI**, **Roche Diagnostics** (Advisor or Review Panel member)

